# ORIGIN: a novel and compact Laser Desorption – Mass Spectrometry system for sensitive *in situ* detection of amino acids on extraterrestrial surfaces

**DOI:** 10.1038/s41598-020-66240-1

**Published:** 2020-06-15

**Authors:** Niels F. W. Ligterink, Valentine Grimaudo, Pavel Moreno-García, Rustam Lukmanov, Marek Tulej, Ingo Leya, Robert Lindner, Peter Wurz, Charles S. Cockell, Pascale Ehrenfreund, Andreas Riedo

**Affiliations:** 1 Center for Space and Habitability, University of Bern, Bern, Switzerland; 2 Space Research and Planetary Sciences, Physics Institute, University of Bern, Bern, Switzerland; 3 Interfacial Electrochemistry Group, Department of Chemistry and Biochemistry, University of Bern, Bern, Switzerland; 4 Life Support and Physical Sciences Instrumentation Section, European Space Agency, ESTEC, Bern, The Netherlands; 5 School of Physics and Astronomy, UK Centre for Astrobiology, University of Edinburgh, Edinburgh, United Kingdom; 6 Laboratory for Astrophysics, Leiden Observatory, Leiden University, Leiden, The Netherlands; 7 Space Policy Institute, George Washington University, 20052 Washington, DC USA

**Keywords:** Astrobiology, Mass spectrometry, Characterization and analytical techniques, Astronomical instrumentation

## Abstract

For the last four decades space exploration missions have searched for molecular life on planetary surfaces beyond Earth. Often pyrolysis gas chromatography mass spectrometry has been used as payload on such space exploration missions. These instruments have relatively low detection sensitivity and their measurements are often undermined by the presence of chloride salts and minerals. Currently, ocean worlds in the outer Solar System, such as the icy moons Europa and Enceladus, represent potentially habitable environments and are therefore prime targets for the search for biosignatures. For future space exploration missions, novel measurement concepts, capable of detecting low concentrations of biomolecules with significantly improved sensitivity and specificity are required. Here we report on a novel analytical technique for the detection of extremely low concentrations of amino acids using ORIGIN, a compact and lightweight laser desorption ionization – mass spectrometer designed and developed for *in situ* space exploration missions. The identified unique mass fragmentation patterns of amino acids coupled to a multi-position laser scan, allows for a robust identification and quantification of amino acids. With a detection limit of a few fmol mm^−2^, and the possibility for sub-fmol detection sensitivity, this measurement technique excels current space exploration systems by three orders of magnitude. Moreover, our detection method is not affected by chemical alterations through surface minerals and/or salts, such as NaCl that is expected to be present at the percent level on ocean worlds. Our results demonstrate that ORIGIN is a promising instrument for the detection of signatures of life and ready for upcoming space missions, such as the Europa Lander.

## Introduction

The detection of signatures of life, past or present, on Solar System objects beyond Earth is of major importance for a better understanding on the presence of life in the universe and how it emerges. Habitability can be traced through several parameters^1,2^, but in particular the detection of biomolecules such as amino acids, lipids and nucleobases on the surfaces of planets and moons are promising indicators for the presence of life. However, their unambiguous detection is extremely challenging and depends on various parameters. Past space exploration missions have focused on detecting biomolecules on Mars and Saturn’s moon Titan^3–5^, albeit without success. Under the premise that life exists or has existed on extraterrestrial bodies, current instruments, such as pyrolysis gas chromatography–mass spectrometry (pyr GC-MS), struggle with the detection of the biosignatures, partly due to the presence of (chloride) salts and minerals^6–11^. To continue the search for molecular biosignatures^1,2^, novel and robust life detection instruments and measurement techniques are important.

Two main candidates for the search of biosignatures in our Solar System are Europa and Enceladus, moons of Jupiter and Saturn, respectively. Previous studies found that these objects have mineral- and organic-rich subsurface oceans^12–15^. On their sea beds, hydrothermal vents could be present^16^. Similar to Earth, such environments represent promising habitats for life, since all ingredients exist for life to emerge^17^. Therefore, these so-called ocean-worlds are prime targets for future space exploration missions devoted to the search for extraterrestrial life^18–20^. Landers are the most promising spacecraft that are able to investigate their surfaces for the presence of biosignatures, in particular biomolecules^20,21^. Such molecules can be brought up from the subsurface oceans and survive within the icy surfaces for millions of years^22^.

Laser Desorption Ionization Mass Spectrometry (LDI-MS) is a powerful tool for the analysis of molecules and allows measurements in various ways^23,24^. In its simplest form, LDI-MS instruments desorb material directly from a surface and ionize it with a single laser pulse, followed by the detection of the ions using a mass analyzer. LDI-MS has a number of advantages over existing space instruments. Due to its high sensitivity, only very low concentrations of analyte are required for a successful detection. LDI-MS instruments can be more compact, requires no consumables, such as carrier gases, and can operate without an extraction furnace, which all affect the dimensions, weight, and power consumption of the instrument; all stringent requirements for space instruments. Furthermore, we show that the detection of biomolecules is not affected by contaminants, such as chloride salts, which in contrast seriously limits the performance of current pyr GC-MS systems^8^. In recent years, various groups started working on LDI detection methods for space instrumentation^25–29^ and a LDI- Quadrupole Mass Spectrometer (LDI-QMS) is part of the Mars Organic Molecule Analyzer (MOMA) suite on the upcoming ExoMars rover^30,31^.

In this paper we present a novel measurement protocol using a compact LDI-MS system, called ORIGIN (ORganics Information Gathering INstrument) for the sensitive detection, identification, and quantification of amino acids. The system consists of a miniature time-of-flight mass spectrometer, developed for *in situ* detection of biomolecules on Solar System bodies, and a nanosecond laser system that desorbs and ionizes surface material with a single laser pulse. Laser desorption studies conducted on pure amino acids allowed the identification of their unique fragmentation patterns under the applied laser desorption conditions. This not only enables the identification of amino acids in more complex mixtures, but also allows their quantification. By careful monitoring specific major and minor biomolecule fragments, surface concentrations as low as a few fmol mm^−2^ can be detected. The results presented here are discussed in light of the requirements for upcoming space missions from ESA and NASA for the investigation of ocean worlds, such as Jupiter’s moon Europa, planned to be realised beyond 2020.

## Results

The ORIGIN system consists of a miniature reflectron time-of-flight mass spectrometer (RToF-MS, 160 mm × Ø 60 mm, m/Δm ≈ 1’000)^32^ that has a nanosecond pulsed laser system as an ion source. The mass analyzer is axis symmetric with a central hole at the entrance and exit. The nanosecond laser pulses (τ ≈ 3 ns, λ = 266 nm) are guided through a beam expander and via various mirrors to the focusing lens. The laser pulses are guided through the mass analyzer to the sample surface, which is positioned below the mass analyzer. Importantly, the sample is positioned slightly out of focus, i.e., the focal point is about a millimeter below the exit. Below the exit, i.e., below the mass analyzer, a steel sample holder on a X,Z translation stage is placed, where the laser desorbs and ionizes the material. Only cations can enter the ion optical system of the mass analyzer and are guided to the micro channel plate (MCP) detector system^33^. For every laser pulse a full ToF spectrum is recorded, which is converted to a mass spectrum (see Sect. 4.1 for a full description of the system). The sample, i.e., organic films of amino acids, are prepared in polished cavities (Ø 3 mm) by dropcasting 1 µL amino acid solution with concentrations ranging from 100–1 µM, resulting in average surface concentrations of 14–0.14 pmol mm^−2^ (see Sect. 4.2 for the sample preparation procedure). Each of these produced cavities is sampled at 40 positions, linearly spaced by ~50 µm, with 100 laser shots at each position (see Sect. 4.3 for the measurement protocol). The resulting mass spectra are filtered based on peak signal-to-noise ratios (SNRs) to remove spectra without signal. For the data handling and data analysis we use in-house developed software (see Sect. 4.4 for the analysis protocol)^34^.

### Amino acid detection and identification

**Figure 1:**
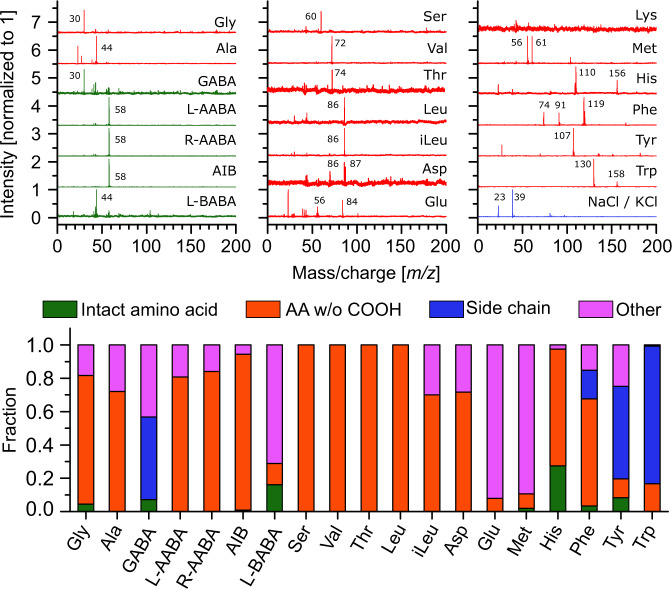
Top: Mass spectra of various biotic (red) and abiotic (green) amino acids (14 pmol mm^−2^ average surface concentration), and of a mixture of NaCl/KCl salt (blue, ~0.7 µg mm^−2^ average surface concentration). See Sect. 4.2 for a list of the abbreviations used for the amino acids. The primary fragments are labelled according to their mass. Bottom: Fragmentation pattern of the measured amino acid spectra (lysine omitted), grouped by masses corresponding to the parent (i.e. intact) molecule (green), the amino acid without its −COOH group (red), the side chain of the amino acid (blue), and other contributions (purple). Signals from −COOH-stripped and side chain masses dominate the amino acid spectra.

The first objective of this study was to show that ORIGIN can detect and identify amino acids and salts placed on a steel surface. Therefore, twenty samples of pure proteinogenic and abiotic amino acid solutions at a concentration of 14 pmol mm^−2^ and an equal parts NaCl/KCl mixture at 0.7 µg mm^−2^ were measured (the full list of amino acids is given in Sect. 4.2). The lowest pulse energy at which a signal was identified was used for the measurement and thus differs for each molecule. The resulting mass spectra are presented in Fig. 1 (top). Except for lysine, all amino acids are detected and display sparse mass fragmentation patterns. Importantly, for the majority of amino acids unique mass fragmentation patterns were observed, although isomers and enantiomers are in some cases difficult to differentiate (e.g. (iso)leucine and L/R-AABA). Figure 1 (bottom) shows the mass fragmentation contributions of the amino acids, divided into parent peak (green), amino acids without the carboxyl (−COOH, 45 amu, red) group, amino acid side chains (amino acid minus 74 amu, blue) and “other” (purple). In the first three groups also (de)protonated fragments are included. It can be seen that the fragmentation patterns are dominated by -COOH stripped and side chain masses. The signal of the NaCl/KCl mixture is dominated by Na^+^ and K^+^ ions and clusters of X^+^(YCl), where X and Y can be Na or K.

### Multi-position scan and quantification of organic film material

**Figure 2:**
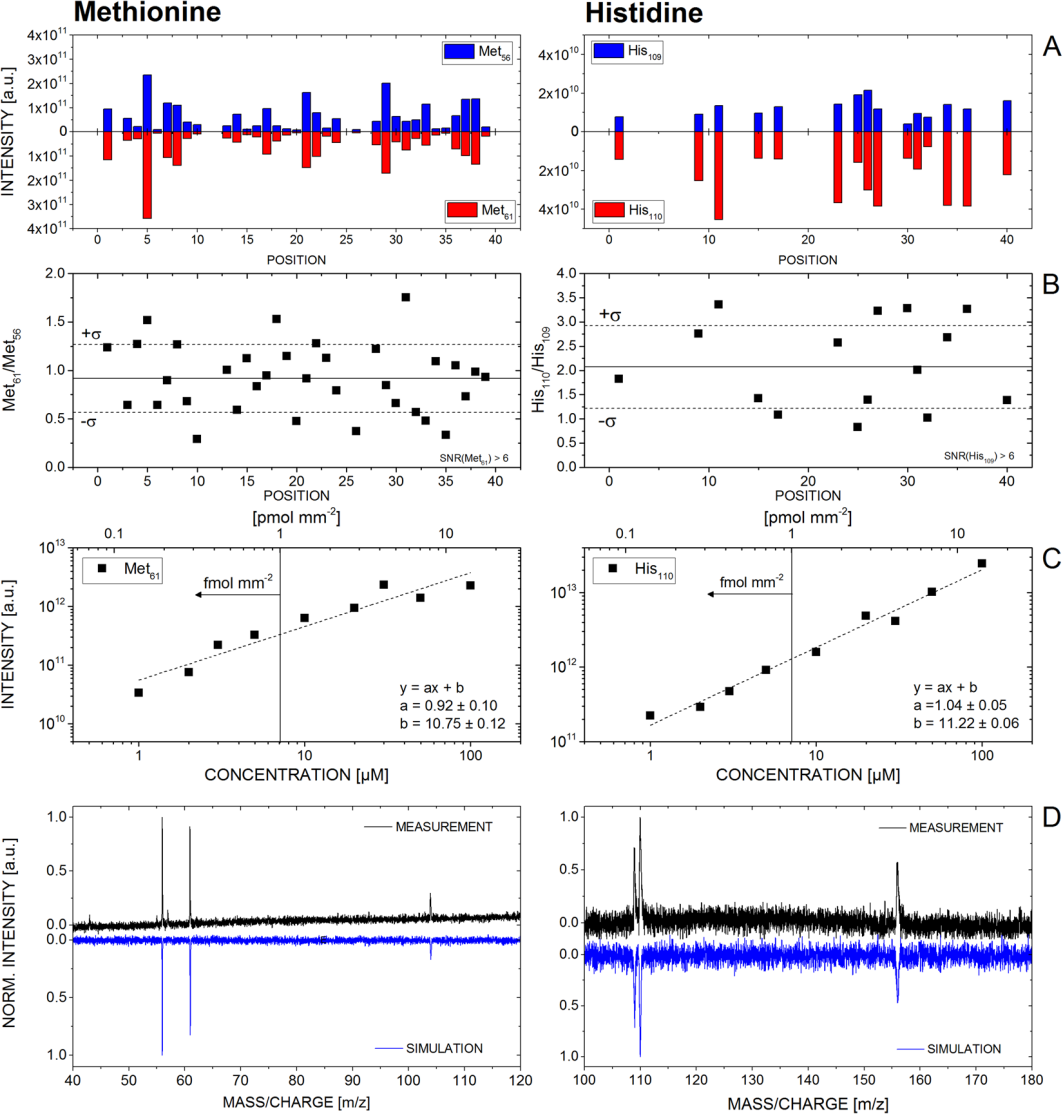
Results of measurements of the amino acids methionine (left) and histidine (right). (**A**) Measured signal intensities of mass 56 and 61 of methionine and 109 and 110 of histidine, at each of the forty positions per scan. Empty positions have signals below a signal-to-noise ratio of six, which are omitted. (**B**) Ratios between the corresponding mass fragments at each of the forty positions. The solid line indicates the mean ratio from the co-added data of all positions. The dashed lines give the 1σ standard deviation of the mean of the measured data. (**C**) Intensities measured as a function of average surface concentration for concentrations in the range of 0.14–14 pmol mm^−2^ (1–100 µM solution concentration), including linear regression coefficients. (**D**) Synthetic mass spectra (blue) compared to the measured data (black).

In Fig. 2, measurements of the amino acids methionine and histidine are highlighted. In panel (A) the intensity of two fragment masses at each of the forty analysed positions is shown, for an average surface concentration of 14 pmol mm^−2^. Signals that are below a SNR of six are excluded. Intensity variations up to an order of magnitude are seen from position to position, which is due to concentration gradients in, and non-uniformity of, the organic film. This clearly demonstrates that the quantification of dried amino acid films using only one spot analysis is highly unreliable. The multi-position measurement protocol circumvents this problem by accumulating signal over the entire diameter of the sample cavity, covering low and high organic film concentrations and therefore effectively averaging the signal. In panel B) the ratios of the two amino acid fragments at each position are displayed and compared to the average ratio obtained from co-added data. Most of the individual ratios are within the 1σ standard deviation obtained from the accumulated data, showing that there is minimal deviation from spot to spot and demonstrating that the fragmentation pattern is quite uniform. In panel C) the accumulated intensities of a single mass peak as a function of the average surface concentration, which ranged from 0.14–14 pmol mm^−2^, are displayed. The data show a fitted linear correlation between signal intensity and surface concentration. From the obtained linear correlation one can conclude that the accumulation of spectra from multiple positions represents a simple and robust procedure for the quantification of amino acid concentrations. The bottom panels depict simulated spectra using the established fragmentation patterns of methionine and histidine, which agree well with the measured data and reproduce the mass spectra well. Similar to spectroscopic measurements where e.g., molecules are identified through their corresponding unique spectroscopic patterns, such a software routine is of high interest to current experiments as amino acids may be identified and quantified in more complex mixtures, including additional contaminates.

### Analysis of amino acid mixture

**Figure 3:**
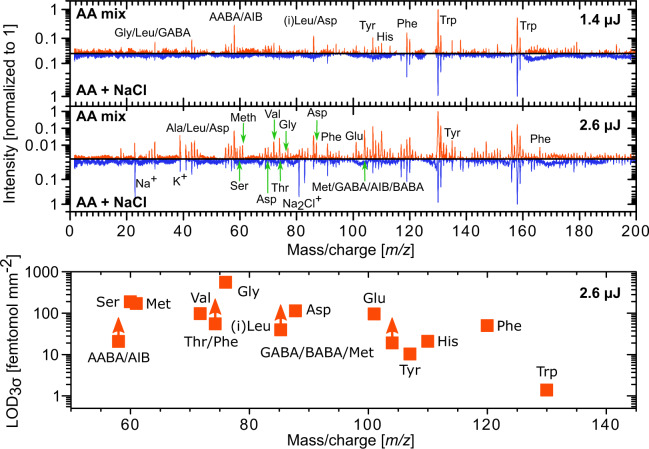
Top: Measured spectra of a mixture of 20 amino acids (0.7 pmol mm^−2^ per amino acid, red) and the same mixture with added NaCl (~0.7 µg mm^−2^, blue). The mixtures were measured with pulse energies of 1.4 (upper panel) and 2.6 µJ (middle panel). Out of the 20 amino acids, eleven can directly be identified from their unique mass features. Bottom panel: 3σ limits of detection (LOD_3σ_) calculated from the 2.6 µJ measurement. Lower limits are given for signals that are clearly detected but have contributions from multiple amino acids.

To test the performance of the system on complex samples, measurements were performed using mixtures containing all twenty amino acids (0.7 pmol mm^−2^ each) with and without added NaCl (~0.7 µg mm^−2^). NaCl is added to simulate measurement conditions on icy moons, which are known to have large amounts of salt embedded in the ice. Figure 3 shows the results for scans at two applied pulse energies (1.4 and 2.6 µJ). The signals are clearly visible and the signal size increases with increasing pulse energy. Except for lysine, mass fragments of all amino acids are clearly visible in the 2.6 µJ measurement. However, not all signals can be unequivocally assigned to a single amino acid, since some of the mass fragments overlap. For example, this hinders the unambiguous identification of alanine just based on the *m/z* = 44 signal. When NaCl is added, the amino acid can still be identified via the mass fragments, however the signal intensities are observed to be lower. Sodium chloride is mostly UV transparent and thus the laser pulse will interact with the amino acids. Therefore, it is likely that the salt crust reduces desorption and/or ionization of the amino acids and higher laser pulse energies may be required. The bottom panel of Fig. 3 shows for the amino acids detected in the 2.6 µJ pulse campaign the 3σ limit of detection (LOD_3σ_, see Sect. 4.5). The high SNR of the mass fragments makes it possible to detect many of the amino acids at surface concentrations below 100 fmol mm^−2^, and in the case of tryptophan even down to 1 fmol mm^−2^.

## Discussion

In this work, we demonstrate that the compact and lightweight ORIGIN system has the measurement capabilities for the identification and quantification of amino acids. With a LOD_3σ_ down to a few fmol mm^−2^ and limit of quantification (LOQ) in the order of 100 fmol mm^−2^, it excels the LDI-QMS instrument (LOD_3σ_ ≤ 1 pmol mm^−2^), which is part of the MOMA suite^30^ on the ExoMars rover, by 1–3 orders of magnitude. Amino acid identification in mixtures is possible, although for some species a deconvolution procedure for studying the fragmentation pattern is needed to distinguish overlapping mass contributions. The need for a deconvolution step will be even more important for real samples, which may contain other organic molecules. It is important to note that the detection sensitivity of our system is currently not set up to maximum sensitivity. For example, the sensitivity can still be increased by ramping the voltage potential over the MCP detector stack^33,35^. A voltage increase over the MCP stack allows the generation of more electrons per single incoming ion and therefore produces an increased recorded signal. In the current setup, the detector gain can be increased by a factor of 32. Furthermore, the multi-position scan method used for the quantification of the organic film material can also be used to increase overall sensitivity by studying more positions on the sample, similar to previous studies conducting ablation campaigns of solids samples^36^. Scanning more positions allows for the direct increase of the SNR and consequently the detection sensitivity. With both methods, the detection sensitivity of the system can be significantly enhanced and is expected to reach sub-fmol mm^−2^ levels.

Besides its high detection sensitivity, ORIGIN has a number of advantages over existing systems for the detection of amino acids in space. For example, with ORIGIN we see no significant chemical reactions between amino acids and chloride-containing compounds, as reported in pyr GC-MS systems^8^. A decrease in amino acid signal intensities is seen, probably caused by NaCl reducing desorption and/or ionization, but in contrast to pyr GC-MS measurements, amino acids remain detectable with ORIGIN. In addition, the presence of NaCl or any other species at high concentration does not saturate the detector thanks to its large dynamic range of up to eight orders of magnitude^33^. The close proximity (~1 mm) of the ToF-MS ion entrance to the sample holder results in a very efficient ion coupling from the desorption plume to the mass spectrometer. Together with a high ion transmission of the time-of-flight analyzer system, low laser pulse energies of only a few µJ can be used in the LDI process, which minimizes molecule fragmentation, enabling a clear identification of biomolecules even in complex mixtures.

A future application of the ORIGIN system could be as payload on a Europa Lander mission, where it can serve as part of the Organic Compositional Analyzer (OCA) to investigate the biomolecular and amino acid content in the icy surface material of Europa. According to the Europa Lander Science Definition report^20^, the OCA needs to be able to detect species at 1 nM in a 1 gram (=1 ml) surface sample. Melting a 1-gram sample and producing an organic film on a surface area similar to the surface area of the cavities used in this work (7.1 mm^2^), results in an average surface concentration of 141 fmol mm^−2^; a concentration easily detectable by ORIGIN. A further requirement in the Europa Lander Science Definition report^20^ is the detection of eight of the following amino acids: Ala, Asp, Glu, His, Leu, Ser, Val, Iva, Gly, β-Ala, GABA, and AIB, ten of which have already been successfully measured by ORIGIN (β-Ala and Iva were not included in the sample). However, securely identifying isomers and enantiomers is currently challenging. Finally, we have demonstrated that the ORIGIN system permits also detecting Na and K and the salts NaCl and KCl. It is very likely that other salts, silicates and metals can also be identified. The results demonstrate that ORIGIN is a promising instrument for the detection of life in upcoming space missions, specifically for the Europa Lander.

## Methods

### Laser desorption mass spectrometry set-up

**Figure 4:**
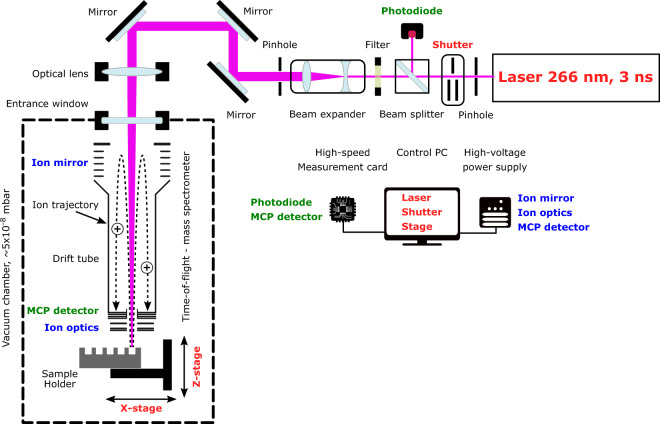
Schematic diagram of the ORIGIN system (not to scale) and of the wiring of the individual components. The mirrors, optical lens, and the beam expander are all placed on stages and manipulators to adjust the laser path.

To facilitate the detection of biomolecules on surfaces of Solar System objects, a novel and compact Laser Desorption/Ionization – Mass Spectrometry (LDI-MS) instrument has been designed and constructed (a schematic depiction is shown in Fig. 4), based on our experiences with existing systems and components^32,37–43^. This system is called ORIGIN (ORganics Information Gathering INstrument). The design is simple and robust, which establishes LDI-MS as a rugged instrument for *in situ* space exploration missions, while at the same time being lightweight and compact.

A Q-switched Nd:YAG laser (Quantel Brio, France) is used for desorption and ionization of sample material (pulse width τ ≈ 3 ns, wavelength λ = 266 nm, pulse repetition rate = 20 Hz). The laser system is equipped with a beam attenuator module for laser pulse energy adjustment. The system guiding the beam to the mass analyzer, the latter is located in a vacuum chamber, consists of a shutter system, attenuator filters, beam expander, high reflective silver mirrors, and a lens system. The shutter system (SH05, Thorlabs) is integrated directly after the laser system and controls the number of laser pulses for each measurement campaign. Following the shutter, an additional set of attenuation filters is placed in the path of the laser beam to (i) operate the laser system at best performance (the stability of the pulse energy is better at higher laser output) and to (ii) reflect laser light to an ultrafast Si-photodiode (Alphalas, Germany) that triggers the data acquisition system (see below). Laser pulses pass through a beam expander (TECHSPEC Vega, Edmund Optics), which expands the beam to about 15 mm in diameter. An iris installed after the expander system is used to cut out the homogenous inner part of the laser pulse (in terms of energy). Via high reflectivity silver mirrors the cleaned laser pulses are guided towards the focusing lens (f = 300 mm), which is mounted on a Z-stage and which allows, first, to keep the sample at an optimal distance relative to the mass analyzer (around 1 mm) and, second, to adjust the position of the laser focus. Subsequently, the laser light passes through a vacuum entrance window into the vacuum chamber where the miniature mass analyzer is located. Typically, laser pulses with energies ranging from 1.4–3.0 µJ, in increasing steps of 0.4 µJ, are applied in the course of our measuring campaigns. With an optical microscope the circular laser ablation spot size was measured to be around 20 µm. For the desorption measurements the laser is slightly out of focus and based on a simplified geometry we calculate the desorption spot to be about 30 µm in diameter. This method likely underestimates the spot size. With a spot size of 30 µm, an energy density of 0.20–2.35 J cm^−2^ or laser irradiances of 66–142 MW cm^−2^ is achieved in the measurements. Just below the laser entrance window, a miniature (160 mm × Ø 60 mm) reflectron-type time-of-flight mass spectrometer (RToF-MS)^32^ is housed. This RToF-MS system is specifically designed for *in situ* space exploration and capable of achieving a mass resolution at desorption conditions of m/Δm ≥ 1’000 with a dynamic range of up to eight orders of magnitude^33^. Voltages of the ion optical system and the detector system (multichannel plate system, chevron configuration) are set remotely using a high voltage power supply. The detector signal is read out by a high-speed analog-to-digital-converter card (U1084A, Agilent, up to 4 GS s^−1^). A high-speed Si-photodiode is used to trigger the data acquisition. The laser pulses pass along the central axis of the mass analyzer. Underneath the RToF-MS, a sample holder is placed on a X,Z-translation stage (Agilis, Newport). With this stage, samples can be moved relative to the laser spot and distinct positions along the X-axis can be probed, in both cases at micrometer precision. The sample holders are introduced into the vacuum chamber and onto the stage via an entrance port. The entire set-up is operated using in-house written Python software, giving control over the laser system, shutter, and sample stage. Fully automated routines make it possible to scan samples at multiple positions, for a certain number of laser pulses and at various laser powers.

The vacuum chamber is evacuated by a small turbomolecular pump (Pfeiffer TMU 071 P, 80 L N_2_) that is backed by an oil free membrane pump, which results in a typical base pressure of about 5 × 10^−8^ mbar. The vacuum chamber is vented using high purity nitrogen (Alphagas 2, Garbagas) that avoids contaminations during sample exchange. Between the vacuum chamber and pumping system, an electronic shutter valve is placed (VAT), which is closed before changing the sample.

### Sample preparation

To measure amino acids and salts at low concentrations and without contaminants, clean working conditions and materials are mandatory. All samples were prepared in a sterilized flow hood. A stainless steel (416 L) sample holder (Ø 27 mm), which has five equally spaced cavities (0.2 x Ø 3 mm), is used to prepare the samples on and introduce them into the LDI-MS setup. Before being used, the sample holder is carefully cleaned by washing it with isopropanol in an ultrasound bath for 15 minutes. Extensive rinsing with Milli-Q grade water (Milli-Q Gradient, TOC < 5 ppb) is followed by a one-hour bake-out at 80 °C to minimize the water content on the sample holder. Finally, the surface of the sample holder is flame sterilized by a propane/butane burning flame at T > 500 °C.

The following fifteen proteinogenic amino-acids were used in this work: Glycine (Gly), L-Alanine (Ala), L-Serine (Ser), L-Valine (Val), L-Threonine (Thr), L-Leucine (Leu), L-isoLeucine (iLeu), L-Aspartic acid (Asp), L-Glutamine (Glu), L-Lysine (Lys), L-Methionine (Met), L-Histidine (His), L-Phenylalanine (Phe), L-Tyrosine (Tyr) and L-Tryptophan (Trp). In addition, five abiotic amino acids were measured: γ-Aminobutyric acid (GABA), L/R-α-Aminobutyric acid (L/R-AABA), α-aminoisobutyric acid (AIB) and L-β-Aminobutyric acid (L-BABA). All amino acids were purchased from Sigma-Aldrich with a purity of >99%. As salts, NaCl (Roth, >99.5% purity) and KCl (Hanseler, >99.5% purity) were used. For each amino acid, a stock solution of 100 µM in Milli-Q grade water was prepared, which was further diluted to concentrations of 50, 30, 20, 10, 5, 3, 2, and 1 µM. The salt solutions were made at a concentration of 1%wt, matching current measurements of the surface of the icy moon Enceladus^13^ and assumed to be similar for Europa, where Na and K have been detected to originate from the surface^44,45^. During the preparation of the solutions, sterilized Eppendorf tubes (Eppendorf BIOPUR Safe-lock tubes) and pipet tips were used (Eppendorf BIOPUR epT.I.P.S.). To prepare the samples on the sample holder, 1 µL of an amino acid or salt solution is dropcast into a cavity and left to dry, producing an organic film and/or a salt crust. Assuming a uniform distribution, amino acid surface concentrations of 0.14–14 pmol mm^−2^ are achieved ([concentration mol L^−1^ × 1 × 10^−6^ L]/[π × 1.5^2^ mm^2^]). Assuming an average amino acid size of 0.5 by 0.5 nm, this converts to a uniform coverage of just 0.2–21 molecular layers of amino acids. However, in reality, the organic film and salt crust are not uniformly distributed and concentration gradients within the cavity are possible.

### Measurement protocol

After sample preparation, the sample holder is introduced into the set-up and the sample chamber is evacuated overnight to reach a pressure of ~5 × 10^−8^ mbar. Before measurements, the data acquisition system, ion optics including detector system, power supply, and laser are given about 45 minutes to warm up and stabilize. The sample holder is moved so that a sample cavity is just outside the laser focus, at ~1 mm distance relative to the RToF entrance electrode.

For each of the studied amino acids, scans with increasing laser power were made to find the lowest laser irradiance at which desorption and ionization of the molecule of interest takes place. This ensures limited fragmentation of the amino acids. The pulse energy used for the detection of each amino acid is given in Table 1.

**Table 1:** Applied laser pulse energies for the detection of various amino acids.

Amino acid	Laser pulse energy	Amino acid	Laser pulse energy
Glycine	1.8 µJ	Leucine	1.4 µJ
Alanine	1.8 µJ	Isoleucine	1.4 µJ
γ-aminobutryic acid	1.8 µJ	Aspartic acid	1.4 µJ
R-α-aminobutryic acid	1.8 µJ	Glutamine	2.2 µJ
L-α-aminobutyric acid	1.8 µJ	Lysine	3.4 µJ (not detected)
α-aminoisobutyric acid	1.8 µJ	Methionine	1.4 µJ
L-β-aminobutanoic acid	1.8 µJ	Histidine	1.8 µJ
Serine	1.4 µJ	Phenylalanine	1.4 µJ
Valine	1.4 µJ	Tyrosine	1.8 µJ
Threonine	1.8 µJ	Tryptophan	1.4 µJ

The non-uniform distribution of sample material in the cavity can result in misleading data when just a single position is sampled. To minimize this effect, a linear scan that consists of 40 positions with a pitch of 50 µm is conducted, which increase our measurement statistics. Subsequently the data are co-added, which effectively averages out variations due to variable sample material concentrations. Doing so, the measured mass signal intensity corresponds linearly to a specific average surface concentration (see Fig. 2c). After having measured the five sample cavities, mass spectra of the clean sample holder (area between cavities) were taken at similar measurement conditions for the purpose of mass calibration and to identify contamination from the sample holder, if present.

### Data analysis

All data are analysed with in-house developed Matlab software^34^. Time-of-flight spectra are selected for peaks with a specified SNR (usually ≥ 6) or for peaks within a certain time window (=*m/z* window). Subsequently, time-of-flight spectra of each single measurement position are co-added, where after spectra of the 40 positions can be co-added to a single ToF spectrum. The software corrects for time-of-flight shifts caused by surface charging, which can occur when the organic film is thick enough to act as an insulator. The interaction of the laser with the film positively charges the latter, which repels the produced cations and makes them arrive slightly earlier at the detector system. The correction is performed by an autocorrelation correction by selecting a peak, which is present in all the acquired time-of-flight spectra. One spectrum is used as a reference spectrum and all the other spectra are shifted so the peak positions of the selected peaks align. Measurements of the steel sample holder are used for the time-of-flight to mass-to-charge conversion by identifying specific ions ablated from the steel holder. The software also generates various statistics from a mass spectrum, such as peak position, integrated peak area, peak full width at half maximum (FHWM), and peak SNR by dividing the peak area by the integrated noise in a region where no signal appears. Blank measurements of the steel sample holder are conducted in each measurement run and used to determine that masses assigned to an amino acid have no or negligible contributions from steel surface contaminants. Simulated, synthetic mass spectra of measured mass spectra are created with our in-house developed software^34^.

### Limit of detection

The limit of detection (LOD) can be given in various ways. The simplest way is to use surface concentration, as we elaborate in Sect. **4.2** by giving ***C***_***surface***_ = 0.14–14 pmol mm^−2^. This procedure is only applicable if a signal is seen for a particular surface concentration ***C***_**surface**_. If the SNR of a detected amino acid mass fragment is sufficiently high, the limit of detection can be lower. Taking a 3σ SNR as the limit for detection, the LOD for a surface concentration is given as LOD_3σ_ = (3/[*SNR*]_fragment_) × ***C***_**surface**_, where [*SNR*]_fragment_ is the fragment signal-to-noise ratio and ***C***_**surface**_ is the surface concentration of the measurement in question. For example, in the measurements of the mixture of 20 amino acids the ***C***_**surface**_ = 0.7 pmol mm^−2^ per amino acid. For tryptophan, the [*SNR]*_130_ = 1731 for the *m/*z = 130 fragment in the 2.6 µJ scan (see Fig. 3), resulting in LOD_3σ_ = 1.2 fmol mm^−2^.

For a meaningful comparison with the LOD requirements given in the Europa Lander Science definition report (1 nM material in a 1 g, or 1 ml, sample, which corresponds to 1 pmol of material), these numbers need to be converted to average surface coverage. Sublimating the water of this sample and distributing the remaining molecules over the same area as the sample holder cavities, i.e., 7.1 mm^2^, results in a surface concentration of 141 fmol mm^−2^.

It remains important to stress that the sensitivity of the system can easily be improved. For example, sampling 400 positions, instead of 40, of the same surface results in a tenfold increase in signal and would already push the LOD_3σ_ of the system into the sub-fmol mm^−2^ regime. In addition, the gain of the MCP detector can still be increased by a factor of 32.
